# Maternal Attachment Representation and Neurophysiological Processing during the Perception of Infants’ Emotional Expressions

**DOI:** 10.1371/journal.pone.0147294

**Published:** 2016-02-10

**Authors:** Rainer Leyh, Christine Heinisch, Johanna Behringer, Iris Reiner, Gottfried Spangler

**Affiliations:** 1 Department for Developmental and Educational Psychology, University of Erlangen-Nuremberg, Erlangen, Germany; 2 Klinik und Poliklinik für Psychosomatische Medizin und Psychotherapie, Johannes Gutenberg-Universität, Mainz, Germany; University of Udine, ITALY

## Abstract

The perception of infant emotions is an integral part of sensitive caregiving within the mother-child relationship, a maternal ability which develops in mothers during their own attachment history. In this study we address the association between maternal attachment representation and brain activity underlying the perception of infant emotions. Event related potentials (ERPs) of 32 primiparous mothers were assessed during a three stimulus oddball task presenting negative, positive and neutral emotion expressions of infants as target, deviant or standard stimuli. Attachment representation was assessed with the Adult Attachment Interview during pregnancy. Securely attached mothers recognized emotions of infants more accurately than insecurely attached mothers. ERPs yielded amplified N170 amplitudes for insecure mothers when focusing on negative infant emotions. Secure mothers showed enlarged P3 amplitudes to target emotion expressions of infants compared to insecure mothers, especially within conditions with frequent negative infant emotions. In these conditions, P3 latencies were prolonged in insecure mothers. In summary, maternal attachment representation was found associated with brain activity during the perception of infant emotions. This further clarifies psychological mechanisms contributing to maternal sensitivity.

## Introduction

As explained by attachment theory [1] children activate and deactivate the caregiving system of attachment figures through the expression of emotional signals. This is also evident on an automatic subcortical level [2,3].

During the course of development recurrent experiences with the primary caregiver lead to specific intrapsychic organizations of attachment (conceptualized as inner working models of attachment (IWM)), which contribute to the regulation of behavior and emotion [1,4]. Parents differ significantly in their responses to infant needs, based on their own attachment security. Parental attachment security is known to be associated with sensitivity, the ability to perceive the child’s signals, to interpret them correctly and to respond to them appropriately [5,6]. Secure attachment figures provide an experiential context of sensitive caregiving for the child [5,7,8], and there is good empirical evidence for the transmission of attachment security from the parental generation to the child [6,9].

Parents use emotional signals of their children to correctly respond to their children`s needs. A relevant cue for detecting another person’s emotions is the person’s facial expression. Accordingly, face recognition tasks were used to investigate differences in the processing of emotions depending on attachment. Such differences are evident in adults. For example, Maier et al. (2005) reported a lower threshold for the perception of emotions in insecure as compared to secure persons, indicating a higher vigilance to emotional stimuli. A heightened vigilance was also found in anxiously attached persons by Fraley et al. (2006). Interestingly, this study additionally showed that the perception of emotions in anxiously attached persons was less accurate. Finally, by using the startle paradigm, Spangler et al. (2010) presented findings indicating a more negative evaluation of negative infant emotions in parents with a dismissing attachment representation. These findings suggest an important contribution of attachment experiences to the processing of emotions in adults.

When investigating neurophysiological processes of emotional face recognition, different event-related components come into play. First, the N170 has been shown as face sensitive, and to be the processing stage of human faces at which a representation of configural features is generated [10–12]. A further component is the P3 which reflects task characteristics like cognitive demand [13] and task difficulty [14]. The P3 is influenced by stimulus novelty [15] as well as overall level of arousal [16]. The underlying processes are thought to be neural inhibition activities associated with attention allocation and memory processes [15]. Emotional faces elicit neural responses which differ from neutral faces [17]. Moreover, emotional face perception is influenced by the emotional state of the perceiver. Anxious people show higher amplitudes to fearful but also to neutral faces. However, psychopaths show general deficits in emotional face recognition [18]. Taken together, context and previous experiences influence how the brain processes emotional faces—a phenomenon which can be investigated by modulation of event-related components.

From an attachment theory perspective it is assumed that a subject’s intra-psychic organization of attachment influences perception, sensation and interpretation of emotions. On the level of underlying processes this may get obvious on the allocation of attentional resources. Accordingly, event-related components in the response to emotional face stimuli should differ depending on the emotion valence (stimulus characteristic) as well as the attentional resources available for the person. Indeed, by applying a backward-masking paradigm within an ERP experiment, Zhang et al. [19] presented happy, fearful and neutral adult faces and found reduced negative components for avoidant as compared to anxious or secure subjects. They concluded that subjects with avoidant attachment style devote a smaller amount of attentional resources during the initial phase of face processing, are less elaborative in encoding structural information of faces, are more susceptible to the arousal of emotional content and have more difficulties in retrieving semantics of faces.

From the transgenerational transmission perspective, attachment differences represent parents’ sensitive responding to infant signals [6], which includes perception and interpretation of emotion. Appropriate perception and interpretation of emotion is seen as a crucial component of secure Inner Working Model of attachment expressed in adults’ representation of attachment [20] which at the same time is predictive of sensitive behavior to their infants’ signals [6,21]. In an experiment employing a startle paradigm, Spangler et al. [22] found that parents with an insecure as compared to a secure attachment representation exhibited a more negative evaluation of infant emotions indicated by mimic responses. Specifically dismissing as compared to secure parents evaluated negative infant emotions more negative on a sub-conscious level indicated by a higher startle amplitude. This negative evaluation may lead to defensive responses and rejection on the behavioral level associated with low sensitivity.

Fraedrich, Lakatos, and Spangler [23] focused on ERPs in mothers during the perception of infant emotions by presenting positive, negative and neutral facial expressions as well as non-facial stimuli within an oddball paradigm. Dismissing mothers exhibited elevated N170 amplitudes for facial target stimuli within conditions that contained frequent non-facial stimuli. Additionally, secure mothers showed elevated P3 amplitudes to target facial expressions during conditions with frequent non-facial stimuli as compared to conditions with frequent infant faces. In summary, this finding suggests that insecure mothers require more cognitive resources to process infant faces, while secure mothers allocate more attention to infant faces and clearly show a perceptual bias toward social information. The study is limited by the fact that the age of the infants pictured on the stimuli did not match the age of the children of the participating mothers. It remains unclear if the effects are stronger when the experimental stimuli are closer in age to the participants’ own children.

To summarize, initial studies provide evidence of attachment-related differences on various neuropsychological stages of emotion processing, while generating new questions.

Firstly, the stimuli presented in the previous experiments could have been better chosen to match the relevance of the attachment system. While Zhang et al. [19] focused on the perception of adult emotions, Fraedrich et al. [23] presented infant emotions. This is an essential difference from an ethological point of view, as according to K. Lorenz the specific features of infant faces contribute to motivating maternal care [24,25]. However, in the latter group [23] the infants did not match the age of the mother’s infants who participated. Future studies should consider this to increase ecological validity.

Secondly, a variety of attachment measurements was used. Unlike Zhang et al. [19] who assessed attachment styles with self-report measures, Fraedrich et al. [23] used representational methods which analyze the coherence of attachment relevant narratives. However, the gold standard to assess attachment representation is the adult attachment interview (AAI; [26–28]. While each of these methods is based on the Bowlby’s attachment theory, a meta-analysis found small to trivial associations between the AAI, used in this study, and self-report measures [29], which indicates that these methods capture different constructs of attachment. This may result in different associations with emotion perception. Consequently, one aim of this study was to apply a more sensitive method and replicate previously reported findings using the AAI to assess attachment representation.

Finally, as the perception of infant emotions is particularly relevant while infants express emotions, the third aim of this study was to extend previous studies by manipulating the frequency of presenting emotional vs. neutral expressions.

Based on previous findings, we built the following hypotheses:

1. The modulation of the N170 by emotional expression or familiarity has often been discussed, underlying its role in bottom-up processes with limited top-down modulation [12,30,31]. The N170 has been elevated when structural encoding is more difficult, e.g. for inverted faces [11]. In Fraedrich et al.’s [23] study, dismissing mothers exhibited elevated N170 amplitudes for facial target stimuli within conditions that contained frequent non-facial stimuli. They conclude, that the configural encoding of faces may be harder for insecure mothers as they activate more processing resources than secure mothers. Using facial stimuli only, we hypothesize that secure mothers require less cognitive resources in the stage of configural encoding of faces and therefore show smaller N170 amplitudes.
2. Higher P3 amplitudes reflect higher involvement of attention and memory processes, indicating a stronger involvement of top-down processes in addition to encoding processes [15]. Secure mothers are able to focus and allocate more attentional capacities to infant emotional expressions, observed in mother-child interactions in attachment research as well as neurophysiologically in the study of Fraedrich et al. [23]. We therefore expect elevated P3 amplitudes for securely attached mothers when seeing infants’ emotions.
3. It is known from behavioral studies in attachment research that parents with a secure attachment representation are more sensitive to infants’ emotional expressions and needs, seem to be better able to handle infant emotions (Ainsworth et al., 1980; Grossmann et al. (1985). We therefore expect to observe differences to insecure mothers especially in conditions with frequent emotion expressions of infants.

## Methods

### Ethics statement

Ethical approval was granted by the review board of the German Society for Psychology (Deutsche Gesellschaft für Psychologie, approval number: GS02102006DGPS). Each participant provided written informed consent.

### Participants and procedure

Thirty-two German mothers from the Erlangen Partner and Parent Study (EPPS; N = 77; see [32]) agreed to participate in this experiment. The experiment took place between 8 and 16 months after the birth of the participant’s first child (M = 11.1, SD = 1.9). In the last term of their pregnancy, the Adult Attachment Interview (George, Kaplan, & Main, 1985) was conducted. Seven participants were excluded because of left-handedness (N = 4, tested by the German version of the Edinburgh Handedness Inventory, Oldfield, 1971), loss of AAI-data (N = 1), the use of psychopharmacological drugs (N = 1) and an early discontinuation of the experiment (N = 1). Hence, 25 mothers aged between 20 and 36 years (M = 29.8, SD = 4.0) were included in the analysis.

### Methods and design

#### Assessment of attachment representation

The mothers’ attachment representation was assessed by the AAI [33], a semi-structured interview consisting of 18 questions focusing on significant childhood experiences, attachment relevant situations in childhood, the evaluation of these experiences as well as the current relationship to the primary caregivers. Transcripts of these interviews were coded on the basis of Main and Goldwyn’s manual [26]. The evaluation of the narrative coherence, idealization and derogation of parents and/or attachment, as well as current preoccupying anger and passivity of speech results in one of the three main attachment categories: Secure (F), Insecure-Dismissing (Ds), Insecure-Preoccupied (E). The AAI’s reliability and validity is well established [6,34–36].

The AAIs in this sample were conducted using the German version of the protocol [37] by the two of the authors (J.B. and I.R.), reliable AAI coders, and an extensively trained psychology student. The audio-taped and verbatim transcribed interviews were subsequently analysed by two certified coders (including the second author). Coding agreement on 35 cases of the original sample was 80% (κ = .68, p ≤ .001). Agreement between coders on coherence of mind and coherence of transcript were κ = .75 (p ≤ .001) and κ = .70 (p ≤ .01) respectively, indicating high agreement. Disagreements between coders were settled by conference. Each participant was categorized according to the AAI to a secure, insecure-dismissing or insecure-preoccupied attachment. Subjects were classified afterwards as secure or insecure.

#### Experimental stimuli and task

The ERP study included a visual oddball task using pictures of infant faces with positive, neutral and negative emotional expressions as stimuli. The pictures were extracted from video-recordings of infant-mother interactions, when the infants were 9 months old. To prevent ERP differences caused by differences in brightness, all pictures were grey scaled. The pictures were edited in a way that the infant face covered the main part of the picture. A positive stimulus valence was defined as a smiling or laughing expression. A negative valence was defined as a face with a crying or a contorted mouth. A neutral valence was characterized by the absence of positive and negative criteria.

The pictures used in this study stem from a picture set used by Fraedrich et al. (2010) who reported successful validation of the pictures with respect to their valence. The mean valence rating for positive, neutral and negative pictures was 2.26, 3.99, and 6.24 on a nine point scale (with higher values indicating more negative valence). A total of 30 different pictures stemming from 10 different infants were used for the oddball task. One half of them (15 pictures) were used for the conditions with positive targets and the other half (15 pictures) were used for the conditions with negative targets. Each set of 15 pictures consisted of 5 positive, 5 neutral and 5 negative pictures.

Within four pseudo randomized conditions (see Table 1), the three emotional expressions were assigned to one of the three oddball related stimulus types: rare targets (10%), rare deviants (10%) and frequent standards (80%). Participants responded by pressing a button with the right index finger to target stimuli that were either positive or negative emotional expressions. Consequently, the two remaining expressions were either standard or deviant stimuli. An example set up would be: positive as target (10%), neutral as deviant (10%) and negative as standard (80%). Each condition consisted of 50 target, 50 deviant and on average of 450 (SD = 6.43) standard stimuli.

**Table 1:** Experimental design: Oddball conditions.

	Target	Deviant	Standard
1	Positive	Negative	Neutral
2	Positive	Neutral	Negative
3	Negative	Positive	Neutral
4	Negative	Neutral	Positive

All stimuli were presented in the center of a black screen at a viewing distance of 115 cm with subtending 9.38° by 9.38° degree of visual angle. The duration of the oddball stimuli was 500ms with a random black screen interstimulus interval between 800 and 1200ms. The picture presentation was controlled by the experimental software “Presentation” by Neurobehavioral Systems. Responses following target, deviant and standard stimuli were registered in ms.

#### EEG recording and quantification

During the oddball conditions continuous EEG was recorded in an electrically and acoustically shielded cabin using an EEG Recording Cap (EASYCAP, Herrsching-Breitbrunn, Germany) with 16 sintered Ag/AgCl electrodes (AFz, Fz, Cz, Pz, Fp1, Fp2, F3, F4, C3, C4, P3, P4, T7, T8, O1, O2) positioned according to the 10-20-system (Jasper, 1958). To monitor vertical and horizontal eye movements, an electrode was placed below and next to the canthi of the left eye. Bio-signals were recorded with a linked mastoid reference. Impedances were kept below 5kΩ. The EEG-signal was registered with a BrainAmp amplifier (Brain Products GmbH, Munich, Germany), digitized at 250 Hz and stored on a hard disk. Using the Brain Vision Analyzer software (BrainProducts GmbH), the signal was filtered offline with a 0.1–35 Hz band-pass filter and re-referenced to the average. The EEG-signal of the oddball-tasks was segmented from the stimulus onset to 900 ms with a pre-stimulus period of 200 ms for baseline-corrections. Individualized thresholds were used offline to reject artifacts caused by eye blinks, baseline shifts or muscles (rejected trials: M = 18.22%, SD = 10.16). Target, deviant and standard stimuli segments were averaged for each participant and condition. To quantify ERP-components of interest, amplitudes and latencies were measured. Latencies were measured in the time windows of 120–180 ms (N170), 200–400 ms (N2) and 300–600ms (P3). The early N170 amplitude was measured by the mean amplitude of the 40 ms surrounding the peak: 125-165ms (N170). Peak amplitudes were measured for the later components N2 and P3. Analyses of N170 and N2 were performed on occipital electrodes O1 and O2 following Fraedrich et al. [23]. Analyses of P3 included the midline electrodes Fz, Cz and Pz. The Presentation software stored the behavioral responses to stimuli and the reaction time in ms and allowed to identify hits and false alarms.

### Statistical analyses

Separate multivariate analyses were conducted with attachment security (secure vs. insecure) as a between-subject factor. As the number of participants within insecure subgroups was small, insecure-avoidant and insecure-preoccupied subjects were combined into one insecure group. In case of significant effects of attachment security, explorative analyses to test subgroup differences were conducted subsequently and will be reported if subgroups differed significantly. The main focus of analysis was on effects concerning attachment, hence only significant effects including attachment security are reported in detail. In case of significant interactions detailed post-hoc analyses were only conducted for interactions with less than four factors.

Behavioral responses were analyzed in terms of reaction times and hit rates to target stimuli as well as false alarms to deviant or standard stimuli. These were entered into a repeated measurement 3-way MANOVA including the within factors target valence (positive vs. negative) and standard valence (neutral vs. emotional) as well as the between factor attachment security (security vs. insecurity). ERP-amplitudes and latencies were analyzed with repeated measurement MANOVAs including the between subject factor attachment security (secure vs. insecure) and the within factors stimulus type (target, deviant, standard), target valence (positive vs. negative), standard valence (neutral, emotional) and component specific electrode sites.

All original data can be found in the supporting information (S1 Table).

## Results

### Attachment representation

In this sample, 16 out of 25 mothers were classified as securely attached. Five subjects were classified as insecure-avoidant and four as insecure-preoccupied. These were combined into the attachment class “insecure attachment” (N = 9). The distribution of the secure and insecure attachment pattern seems to be representative (see van Ijzendoorn, 1995).

Preliminary analyses were conducted to test for possible influences of maternal age and the infant’s age at data assessment on attachment and neurophysiological measures. Maternal and child age was neither related to maternal attachment, behavioural measures (hit rate, false alarm, reaction time) nor systematically related to the neurophysiological measures (only 2 out of 84 correlations for amplitude scores and only 4 of 84 correlations for latency scores were significant). Therefore, age measures were not included in the following analyses.

### Behavioural data

Regarding reaction times, hit rates, and false alarms a three-way MANOVAs (target valence x standard valence x attachment security) revealed a marginally significant effect of attachment security (F(1,23) = 3.35, p≤.10, η^2^ = .13) on hit rates. On average, insecure mothers (M = 45.0, SD = 5.7) recognized target stimuli to a smaller extent than secure mothers (M = 48.0, SD = 2.6). Although no significant interaction between attachment and target valence was found, the means indicated that the effect specifically applied to negative targets. This was supported by separate ANOVAS for targets with negative and positive valence. Secure mothers recognized more negative targets (M = 48.1, SD = 0.7) than insecure mothers (M = 43.7, SD = 2.5; F(1,23) = 4.55, p≤.05, η^2^ = .17), whereas the hit rates to positive targets did not differ between the two groups (M = 48.0, SD = 1.1 and M = 46.3, SD = 2.0 for securely and insecurely attached mothers, respectively; F(1,23) = .64, n.s.). There were no attachment effects for reaction times and false alarms.

### Event-related potentials

In summary, the five-way MANOVAs (target valence x standard valence x stimulus type x electrode x attachment security) revealed significant attachment-related effects for the N170 and P3 component. Significant attachment-related effects were not found for the N2 component and the N170 latencies and, therefore, are not reported here.

#### N170 amplitude

**Fig 1:**
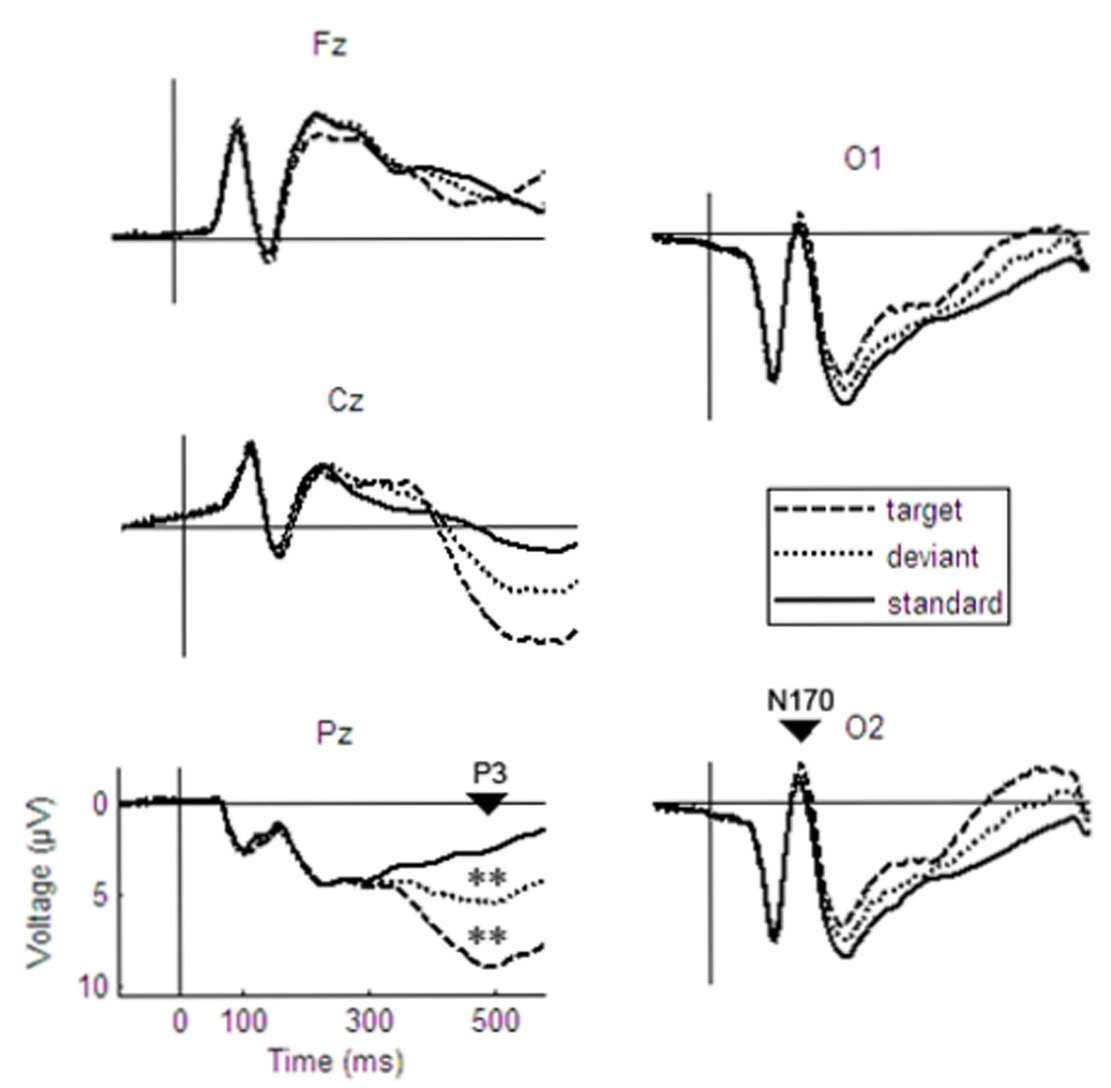
Grand averages of ERPs to target, deviant and standard stimuli for the P3 (left side) and the N170 (right side). ** < .01.

**Fig 2:**
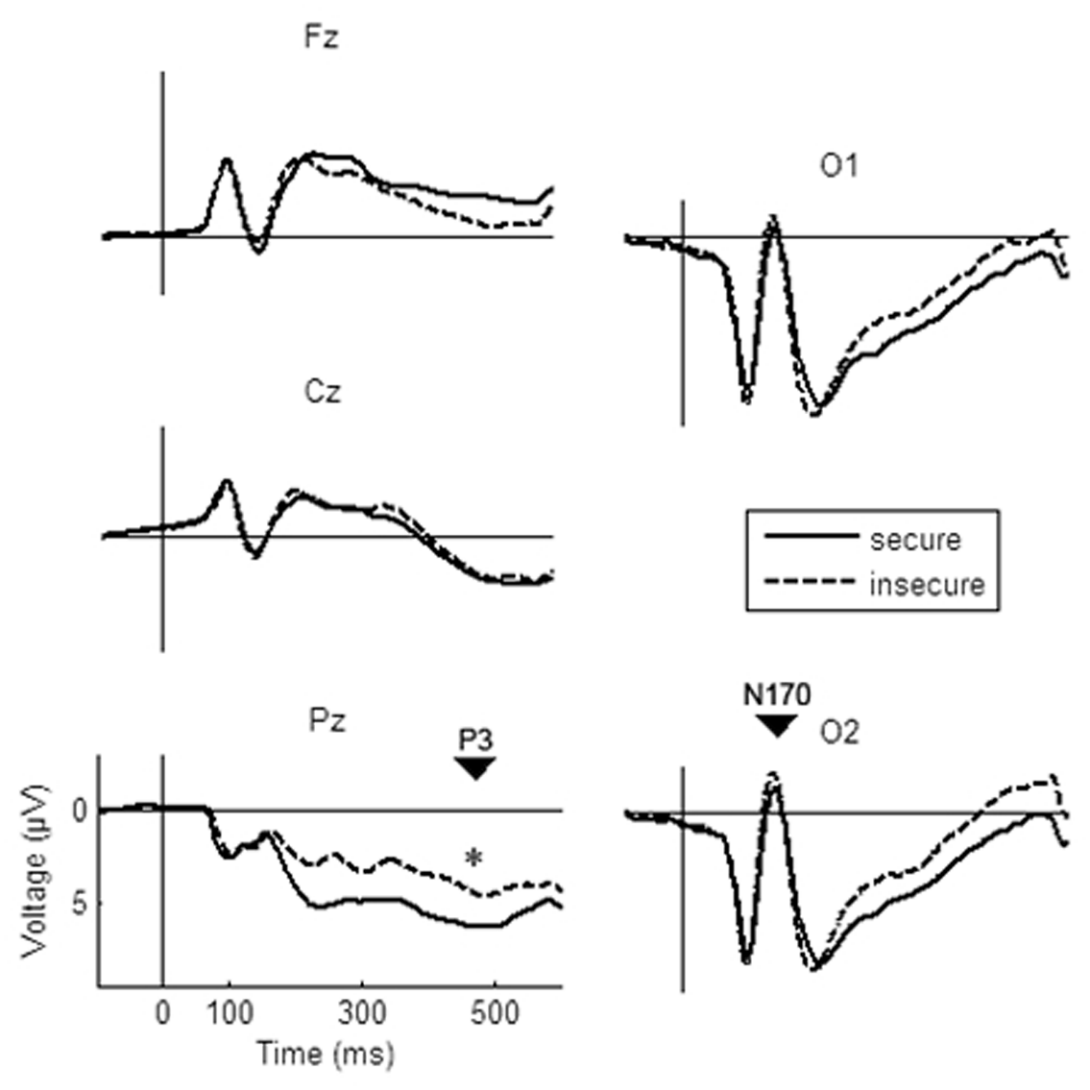
Grand averages for secure versus insecure mothers for P3 (left side) and N170 (right side). * < .05.

**Fig 3:**
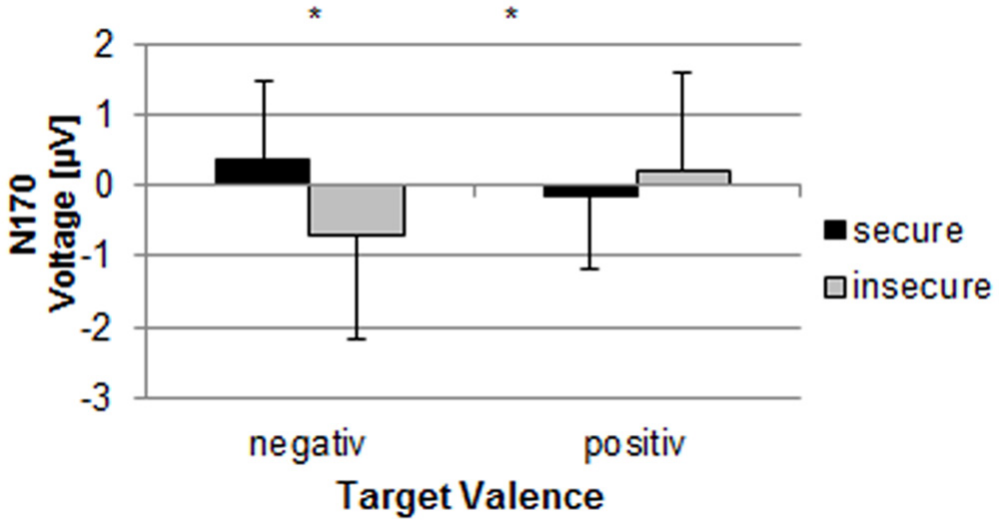
N170 amplitude for secure and insecure mothers during conditions with positive and negative target valence. *: p < .05.

Figs 1 and 2 show the grand average within the N170 time range, which is comparable to existing literature (e.g. [38]. The five-way MANOVA (target valence x standard valence x stimulus type x electrode x attachment security) for the mean amplitudes revealed a significant interaction between target valence and attachment security (F(1,23) = 7.58, p≤.05, η^2^ = .25). Duncan post hoc tests (p < .05) yielded a more negative mean N170-amplitude during conditions with a negative target stimulus in insecure mothers than in secure ones (see Fig 3). Insecure mothers also displayed more negative amplitudes during conditions with a negative target than during conditions with a positive target (p≤.05).

#### P3 amplitude

A two-way electrode (Fz, Cz, Pz) x stimulus type (standard, deviant, target) MANOVA of the P3 amplitude revealed significant main effects for the factors stimulus type (F_(2,22)_ = 84.70, p≤.01, η^2^ = .89) and electrode (F_(2,22)_ = 60.72, p≤.01, η^2^ = .85). Post-hoc ANOVAs revealed the highest P3 amplitudes to target stimuli (F_(1,23)_ = 75.6, p≤.01, η^2^ = .77) and at the Pz electrode (F_(1,23)_ = 112.7, p≤.01, η^2^ = .83), which confirms the typical scalp distribution and stimulus type effect of the P3 described in the literature (e.g. [39] see Fig 1). To reduce the number of electrodes, further analyses focus on the parietal maximum (Pz).

**Fig 4:**
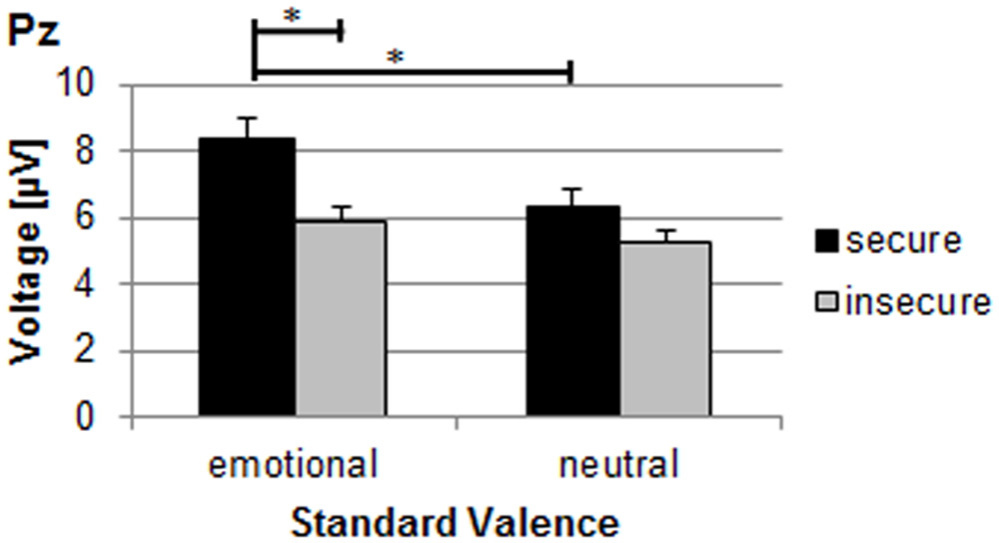
P3 amplitude for secure and insecure mothers during conditions with emotional and neutral standard valence. Emo: conditions with positive and negative standard valences; neut: conditions with neutral standard valence *: p < .05.

The four-way MANOVA (target valence x standard valence x stimulus type x attachment security) for P3 amplitudes resulted in a significant main effect of attachment security (F_(1,23)_ = 7.22, p≤.05, η^2^ = .24) and a significant interaction between attachment security and standard valence (F_(1,23)_ = 7.25, p≤.05, η^2^ = .24). On average, securely attached mothers exhibited a more positive P3-amplitude than did insecure mothers (see Fig 2). Regarding the interaction, simultaneous Duncan post hoc tests revealed that secure mothers showed a greater P3 positivity within conditions containing an emotional standard valence as compared to conditions with neutral standards (p≤.05; see Fi 4), which was not the case for insecure mothers. During emotional standard conditions, the P3 of secure mothers in emotional standard conditions was also higher in amplitude than the P3 of insecure mothers (p≤.05; see Fig 4).

In a subsequent explorative analysis, we analyzed whether the interaction between attachment representation and standard valence for the P3 amplitude can be identified for each stimulus type in the same way. The interaction between attachment security and standard valence turned to be significant only for target stimuli (F_(1,23)_ = 6.12, p≤.05, η^2^ = .21). Simultaneous Duncan post hoc tests revealed the same pattern as did the analysis for all stimuli together. In conditions with emotional standards, secure mothers showed an elevated P3-amplitude due to target stimuli (p≤.05).

#### P3 latency

**Fig 5:**
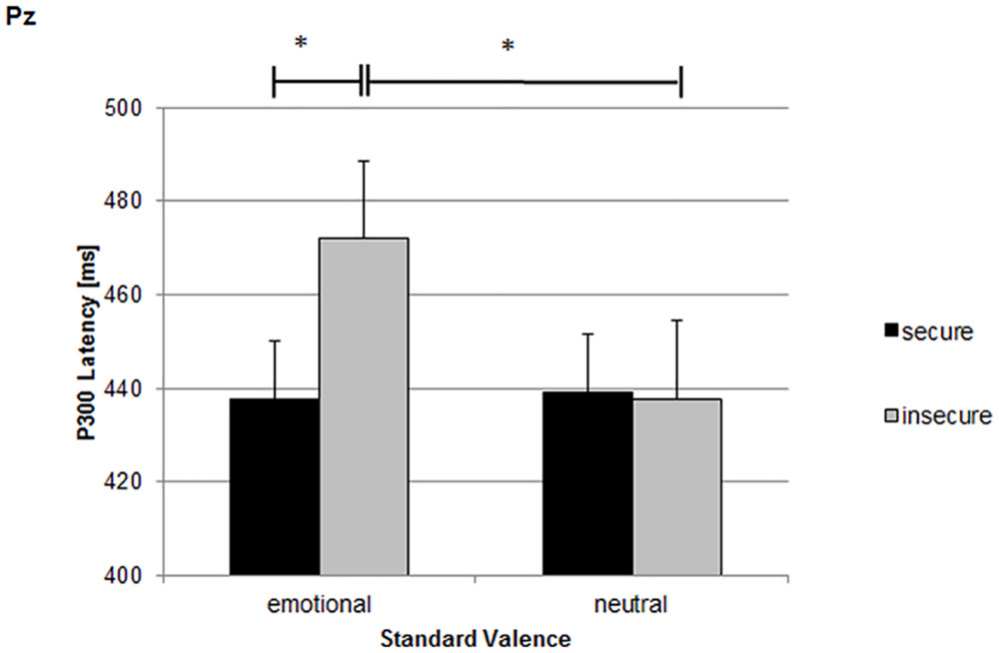
P3 latencies for secure and insecure mothers during conditions with emotional and neutral standard valence. Emo: conditions with positive and negative standard valences; neut: conditions with neutral standard valence. *: p < .05.

To analyse P3 latencies, the Pz activity was included, as the topographic maximum at Pz has been verified before. Focusing on attachment security, the four-way MANVOA (stimulus type x target valence x standard valence x attachment security) revealed a significant interaction between attachment security and standard valence (F_(1,23)_ = 4.98, p≤.05, η^2^ = .18). Simultaneous Duncan post hoc tests showed that insecure mothers had larger latencies in conditions with emotional standards than in conditions with neutral standards (p≤.05) and they exhibited longer latencies during emotional standards than the secure mothers (p≤.05; see Fig 5).

An explorative analysis to test whether the interaction between attachment security and standard valence was evident for each stimulus type again revealed that the interaction between attachment security and standard valence was significant only for targets (F_(1,23)_ = 5.20, p≤.05, η^2^ = .18).

## Discussion

### General discussion

The present study aimed to investigate the early processing of mothers toward infant facial emotion expressions depending on their attachment status by using an emotional perception task. Differences related to attachment security were found on the behavioral level as well as on the neurophysiological level.

On the behavioral level, securely attached mothers detected more infant emotional expressions that were defined as target emotions. This is in line both with theoretical assumptions from attachment theory suggesting a higher ability to recognize emotions and similar findings have been reported by previous studies [23,40]. The additional finding that this difference between securely and insecurely attached mothers was based on infant pictures depicting negative emotional expressions is of particular interest, as in attachment theory, expression of negative emotions is regarded as attachment behavior shown in situations of distress in order get proximity from the caregiver [1] which provides protection, support and emotion regulation. The perception of infant negative emotion is an integral component of parental sensitivity which, on the one hand supports the development of a secure infant-caregiver attachment relationship (Ainsworth et al., 1978; Grossmann et al., 1985), and, on the other hand, is a typical behavioral characteristic of parents with a secure attachment relationship (for a meta-analysis see van IJzendoorn, [6].

With respect to the neurophysiological level, secure and insecure mothers were found to differ in their neural processing during the emotion perception task, consistent with previous studies [19,23]. The neurophysiological findings of the N170 mirror the findings of the behavioral responses. When the target depicted an infant with negative emotions, mothers showed different N170 amplitudes depending on their attachment representations. Specifically, insecurely attached mothers exhibited more negative N170 amplitudes than securely attached mothers after presentation of faces with negative emotions. Fraedrich et al. also found the enhanced N170 in the context of emotion perception in insecure mothers, but only when the target emotional expressions were embedded in frequent non-facial stimuli. By adjusting the task and presenting infants of the same age as the mother’s infants, and by using the AAI as a more sensitive assessment of attachment status, the present study emphasize the specific role of infants’ negative emotions.

At least, two different explanations are possible for this finding. On the one hand, previous studies demonstrated that the amplitude of the N170 is modulated by the participants’ emotional state, including anxiety [41–44,44]. Taking the social psychological tradition of attachment research, anxiety (in addition to avoidance) is conceived as one central dimensions of attachment insecurity [45]. It can therefore be assumed that insecure mothers, at least the preoccupied ones show elevated N170 amplitudes. On the other hand, considering previous studies reporting an augmented N170 negativity for scrambled or inverted faces [46,47], a higher N170 amplitude indicates a higher difficulty of processed stimuli. The finding of an enhanced N170 amplitude in mothers with an insecure attachment representation, when they have to detect negative emotions, could therefore imply that it is more demanding for them to process configural features of infant faces depicting negative emotions.

Differences between secure and insecure mothers were also found regarding amplitude and latency of the P3 component, reflecting differences in attention and memory processes [15] during the perception of infant emotional expressions. P3 amplitudes seem to be closely linked to emotional responses to presented stimuli [48,49]. This indicates that emotional stimuli provoke more attention in the observer. Regarding the main effect of attachment security for the P3 amplitude in the present study, demonstrating an elevated P3 positivity during perception of infant emotional faces in secure as compared to insecure mothers, suggests that secure mothers are more able to allocate attention to infant emotional signals. Fraedrich et al (2010) reported a similar finding when presenting infant emotional faces among by using a non-social-stimulus as a standard stimulus. They postulated a perceptual bias towards social stimuli for secure mothers.

In particular, detailed analyses in this study showed that secure in contrast to insecure mothers showed an increase of the P3 amplitude during conditions with emotional, but not neutral standard stimuli. Thus, not only the emotional valence of the stimuli presented but also the emotional context seems to contribute to attention processes in secure mothers. Findings on the relevance of the emotional context were reported by Doi and Shinohara [44], who found increased P3 amplitudes in mothers when viewing the straight gaze of an unfamiliar child as well as the averted gaze of the own child. Their interpretation was that mothers are highly vigilant to potential threats in the environment. In addition, earlier studies [50,51] showed that the P3 could also be enhanced by the mother’s arousal which is caused by recognizing themselves as the source of attention for an unfamiliar child. For the present study this would imply that the higher P3 amplitudes in secure mothers indicate a state of higher attention when the standard stimulus depicted an emotional but not a neutral face. This indicates that the attention is increased by an emotionally relevant context in secure mothers, while insecure mothers do not adjust resources depending on the emotional context.

The differential effect of the emotional context depending on maternal attachment representation was also obvious for the P3 latency. Insecure mothers exhibited elevated P3 latencies within conditions that contained frequent emotional expressions of infants. P3 latency reflects processing speed during stimulus evaluation [15,52–54]. This suggests that for insecure mothers, the processing of facial indicators of emotions is slowed down by a context of expressed infantile emotions. The effects for the P3 amplitude and the P3 latency taken together, it seems that mothers with an insecure attachment representation are not able to allocate additional attentional resources in an emotional context and, maybe therefore, need more processing time. Nevertheless, considering the lower hit rate in detecting the target emotion, the additional processing time is not sufficient to compensate the lower resources.

The effect of attachment security might indicate that secure mothers invest more cognitive resources in the processing of differences between emotional expressions of infants with minor variations. As reduced P3 amplitudes were also found in tasks with heightened difficulty [14], the smaller amplitude, associated with attachment insecurity, and the longer latency may reflect increased demands in the processing of infant faces for insecure mothers. This might lead to the well-known advantages in the perception of emotions in secure individuals (e.g. [40], as well as, more broadly, to higher social competence [55,56]. In the present study, these differences in neurophysiological processing, indicating heightened attention to infant emotions in mothers with a secure attachment representation, were also reflected in their behavioral responses during the experiment, a heightened hit rate in detected the target emotion. This parallels findings from naturalistic behavioral observations of highly sensitive responding to infants in secure mothers (van Ijzendoorn, 1995).

In this study the response patterns of persons with an insecure attachment representation (dismissing and preoccupied) did not differ. According to Kobak and Sceery (1988) the secure and insecure attachment groups can be arrange along two different dimensions, attachment security and emotional hyper-/deactivation. While both hyper- and deactivating subjects may be included in the secure pattern, the two insecure groups differ with respect to this dimension with de-activation being an essential characteristic of the insecure-dismissing pattern and hyper-activation being typical for the insecure-preoccupied pattern. The findings of this study indicate a main effect of attachment security, independent of the type of the insecure pattern. Thus processing of infant emotions on a neurophysiological level in mothers seems to be more associated with experiences of efficient emotional regulation within the primary caregivers rather than with dispositions of emotional activation.

### Limitations

Although the neurophysiological approach applied in this study revealed new insights into attachment-related differences in mothers perceiving infant emotions, several limitations should be noted. First of all, the sample size of 25 mothers resulted in small groups of insecurely-avoidant and preoccupied attached subjects. Therefore, they had to be combined to one insecure group, even though the two insecure groups may differ regarding emotion relevant processes [4]. However, explorative analyses of differences between the insecure groups yielded no significant effects.

Moreover, by presenting pictures of unfamiliar infants during the experiment our study focused on the perception of infant emotion expression in general. However, familiarity has a significant impact on processing of faces [38,57,58]. Several studies provided evidence that viewing one´s own infant’s face in contrast to unfamiliar infants exhibits a distinctive pattern of brain activation, including the PFC and emotion and theory- of mind related areas [59–64]. It is unclear, if insecure mothers perceive faces of infants differently from secure mothers per se or if seeing their own child would alter face perception even more depending on attachment style. Initial hints at this can be taken from Doi and Shinohara [44], who found different activation patterns when seeing the own or an unfamiliar child with different gaze directions. However, this study again lacks the information about the mothers’ attachment style. Therefore, future studies should focus on neuropsychological, attachment-related differences in the perception of emotions of one´s own children.

### Conclusion

In summary, these results further uncover the underlying neurophysiological processes that contribute to a higher sensitivity toward infants of secure as compared to insecure mothers [6,21]. Within the concept of parental sensitivity [5], there is an emphasis on the correct perception and interpretation of emotional states and needs of children. Facial expressions provide reliable information for drawing conclusions about the underlying emotional state [52,65–67]. This makes it very important for parents to recognize infant emotions, which is the precondition for an adequate and sensitive response. Investigating brain activity, our results show that that the attachment representation, the result of the own attachment history [6], affects the configural processing of infant faces, attention and memory processes, as well as processing speed during the perception of infant emotions. This finding supports the view that a mother’s brain processes infants’ faces differently depending on their own experience. Moreover, it contributes to explaining the neurophysiological basis of processes linked to the transgenerational transmission of attachment security.

## Supporting Information

S1 Table Original data can be found in the supporting information. Variables are described as the following: Analyzed amplitude (N170, N200, P300); t = target, st = standard, p = positive, n/neg = negative, nt = neutral; Electrode position; LAT = Latency, AMP = Amplitude. VP indicates the subject code. No identifying information is contained within the datafile.
(SAV)
